# Artificial Neural Network Analyzing Wearable Device Gait Data for Identifying Patients With Stroke Unable to Return to Work

**DOI:** 10.3389/fneur.2021.650542

**Published:** 2021-05-19

**Authors:** Marco Iosa, Edda Capodaglio, Silvia Pelà, Benedetta Persechino, Giovanni Morone, Gabriella Antonucci, Stefano Paolucci, Monica Panigazzi

**Affiliations:** ^1^Department of Psychology, Sapienza University of Rome, Rome, Italy; ^2^Scientific Institute for Research, Hospitalization and Healthcare (IRCCS) Santa Lucia Foundation, Rome, Italy; ^3^Occupational Therapy and Ergonomics Unit, Istituti Clinici Scientifici Maugeri IRCSS, Pavia, Italy; ^4^Department of Occupational and Environmental Medicine, Epidemiology and Hygiene, Italian Workers' Compensation Authority (INAIL), Rome, Italy; ^5^Occupational Therapy and Ergonomics Unit, Istituti Clinici Scientifici Maugeri IRCSS, Montescano, Italy

**Keywords:** neurorehabiliation, long-term disability, occupational medicine, psychometrics, walking, artificial intelligence, machine learning

## Abstract

A potential dramatic effect of long-term disability due to stroke is the inability to return to work. An accurate prognosis and the identification of the parameters inflating the possibility of return to work after neurorehabilitation are crucial. Many factors may influence it, such as mobility and, in particular, walking ability. In this pilot study, two emerging technologies have been combined with the aim of developing a prognostic tool for identifying patients able to return to work: a wearable inertial measurement unit for gait analysis and an artificial neural network (ANN). Compared with more conventional statistics, the ANN showed a higher accuracy in identifying patients with respect to healthy subjects (90.9 vs. 75.8%) and also in identifying the subjects unable to return to work (93.9 vs. 81.8%). In this last analysis, the duration of double support phase resulted the most important input of the ANN. The potentiality of the ANN, developed also in other fields such as marketing on social networks, could allow a powerful support for clinicians that today should manage a large amount of instrumentally recorded parameters in patients with stroke.

## Introduction

In a complex and fast-changing environment in which a growing amount of data is everyday collected, there is a need to find patterns and connections to make better decisions at every turn. Artificial neural networks (ANNs) are increasingly being used with these purposes. An artificial neural network is a machine learning algorithm inspired on the brain biological neural networks, with an artificial intelligence inspired by the human one (1). Among all the artificial intelligences, ANNs are a type of model for machine learning widely used, for example, in social networks to define customer profiles and discover their preferences, hence optimizing marketing campaigns.

In the scientific healthcare field, there is a growing amount of electronic data, deriving from sensors and electronic clinical sheets, that may favor a medical outcome analysis, for example, for predicting the length of a hospital stay or the risk of fall associated to a walking patient. However, given the wide amount of data, there is a need for automatic analysis that could have the ability to discover complex relationships in the data and generate accurate performing predictive models. In this field, ANNs are becoming relatively competitive to prognostic regressions and other conventional statistical models (2).

An artificial neural network is a non-linear data computational model consisting of input and output layers plus one or more hidden layers. The connections between neurons in each layer have associated weights, which are iteratively adjusted by the training algorithm to minimize error and provide accurate predictions (3).

For patients with stroke, artificial neural networks have been used as models for screening (4), risk identification (5), or as a prognostic tool (6). Lee and colleagues were pioneers in using an ANN with an accuracy of about 80% in identifying movement disorders from spatial parameters obtained by video analysis of gait (7). Scheffer and Cloete had the intuition of the potentialities of combining two emerging technologies: an artificial neural network and inertial motion capture (8). In their study, the ANN was able to correctly classify patients with stroke in 99.4% of cases with respect to healthy subjects starting from the data of an inertial measurement unit (IMU). So, they suggested the usability of the ANN and IMU for planning gait rehabilitation therapy and monitoring its outcomes in stroke. For years, gait analysis was performed using complex stereophotogrammetric systems requiring large economic and temporal resources, whereas now there is a wide diffusion of more simply (despite less informative) wireless inertial sensors that allow to compute the spatiotemporal parameters of gait and trunk kinematics during walking (9–11). Among the information provided by IMUs, the upright gait stability has been associated to the risk of fall (12), and walking speed resulted an important prognostic factor of functional recovery (13), community mobility, and quality of life in patients with stroke (14).

We have recently highlighted that in subjects in which stroke occurred in their working age, the long-term disability affects the possibility to return to work (RTW) and, in turn, the quality of life after discharge from a rehabilitation hospital (15). In fact, psychological and economic problems can be related to the impossibility to return to work after stroke, as it occurs in about 80% of workers suddenly impaired by stroke (16). This is a dramatic percentage, especially considering that the mean age of stroke onset is decreasing, and, in Western countries, the retirement age is increasing, leading to an increment of the incidence of stroke during the working age (17). The return to work may depend on many several cognitive and motor factors, strictly intertwined with each other; among these is the independence in daily living activities including walking (15).

The aim of this pilot study was to use an ANN for analyzing the data of a wireless inertial system for gait assessment to evaluate the possibility to return to work after stroke.

## Materials and Methods

### Participants

Thirty-three subjects were enrolled in this study, all of them in working age (between 18 and 66 years), 17 healthy subjects and 16 patients with a diagnosis of stroke in chronic phase (7 with left hemiparesis and 9 with right hemiparesis). Exclusion criteria included cognitive impairments with Mini-Mental State Examination < 24, severe unilateral spatial neglect, and severe comorbidities. The age of patients ranged between 21 and 66 years old (mean age: 54.6 ± 13.7 years), whereas that of healthy subjects ranged between 22 and 63 years old (mean age: 45.7 ± 13.4 years, not significantly different from that of patients, *p* > 0.05). Ten patients did not return to work at the moment of the evaluation. Independent Local Ethical Committee approved the study, and all the participants signed the informed consent.

### Gait Analysis

Gait data were acquired by means of a wearable inertial measurement unit endowed with a triaxial accelerometer, a triaxial gyroscope, and a magnetometer (G-Walk, BTS, Padua). The device was placed at the level of the sacral vertebras S1–S2 embedded into an ergonomic waist belt. This wearable IMU was connected to a portable computer via Bluetooth. The sample frequency of recording was 100 Hz. Subjects were asked to walk along a linear pathway of 10 m from a starting to a stop line. During walking, the IMU recorded lower trunk accelerations and angular velocities (respectively, along and around the anterior–posterior, laterolateral, and craniocaudal body axis), estimating from these signals the gait temporal and angular parameters. Given the information about the path length, the IMU software also computed the walking speed and step lengths (18). The temporal variables extracted by the IMU and acquired by the ANN were as follows: the cadence of steps, the stance, the swing, the single support, and the first and second double support gait phases. The trunk kinematics variables extracted by the IMU and acquired by the ANN were as follows: the range of motion (ROM) of the trunk tilt, obliquity, and rotation (also definable as trunk rotations around the latero-lateral, antero-posterior, and cranio-caudal axes, respectively, or as pitch, roll, and yaw). Despite from a theoretical point of view right and left strides are equal in a reliable walk, the measured parameters often do not, so for all the above parameters, but cadence, we computed the absolute mean value between left and right strides and their differences.

### Artificial Neural Network

**Figure 1:**
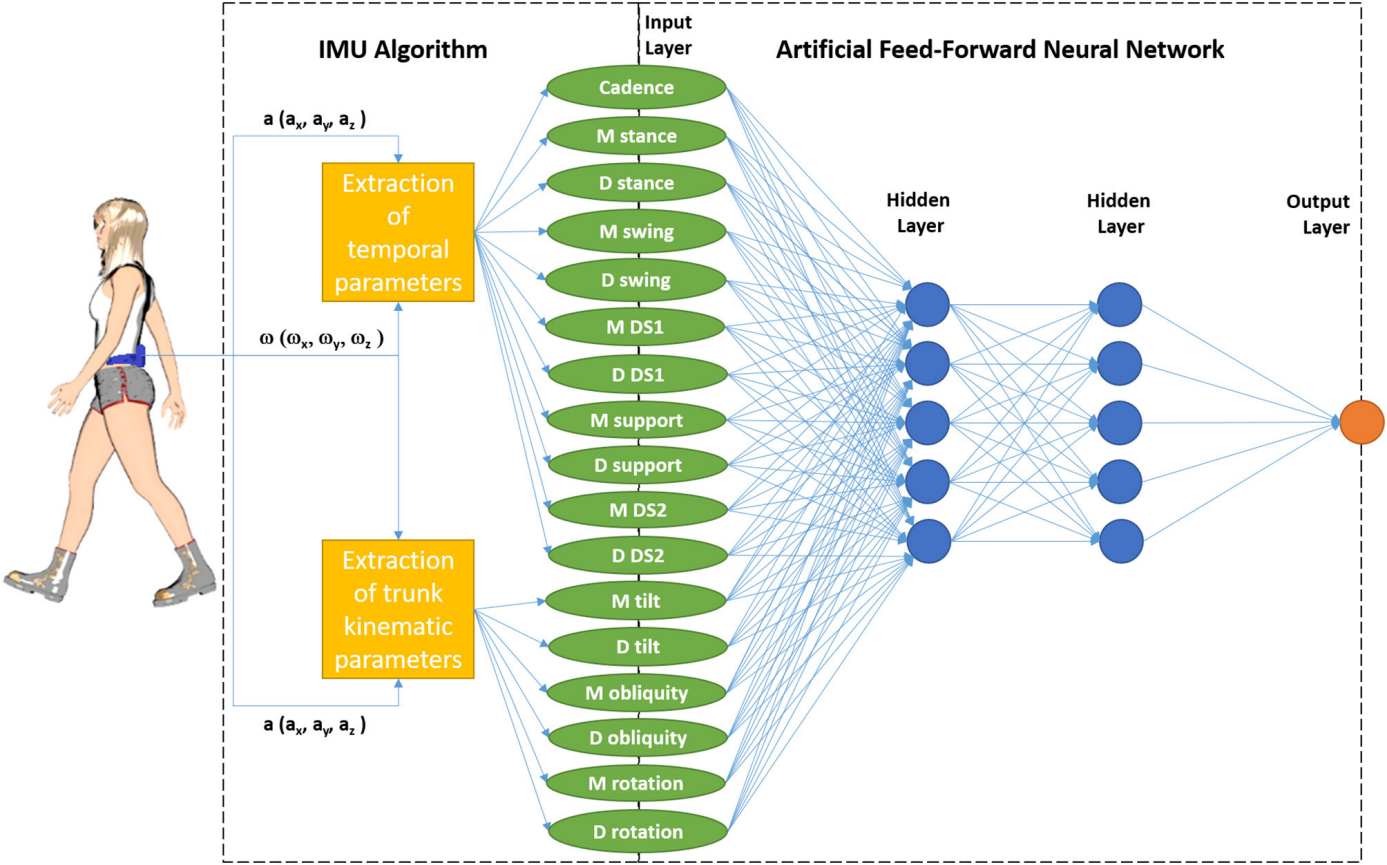
Schematic representation of the combination of Inertial Measurement Unit (IMU, measuring triaxial acceleration a, and triaxial angular speed ω) and its software with the feedforward neural network formed by an input layer (M stands for mean values between right and left strides, and D for their difference, DS 1 and 2 for first and second double support phases; support is the phase with only one foot on the ground), two hidden layers, and an output layer (which represents the identification of patients in the first analysis and the identification of patients who did not return to work in the second analysis).

We designed an artificial neural network working on the basis of a multilayer perceptron procedure. The ANN was formed by four layers: the input layer from which the above listed 17 variables extracted by the IMU software were entered, 2 hidden layers of 5 elements each, and a final output layer (Figure 1). The architecture of our ANN was that of a feedforward neural network (FFNN), with data moving in only one direction, from the input nodes through the two hidden layers to the output nodes (3). The activation functions for all units in the hidden layers and that for the output layer were both a hyperbolic tangent. The chosen computational procedure was based on an online training (details: initial learning = 1.2; lower learning = 0.001, learning epochs = 10, momentum = 0.9 interval center = 0, interval offset = ±0.5, memsize = 1,000, steps without error = 1, error change = 0.0001, error ratio = 0.001). The ANN was developed using the IBM SPSS Neural Networks module of IBM SPSS Statistic, version 23.

Firstly, we tested this ANN on its capacity to identify patients with respect to age-matched healthy subjects. Then, we tested the ANN on the identification of patients who did not return to work on the entire sample (patients and healthy subjects) and finally on their identification only among patients. It means that the dependent variable was categorical, and the ANN worked to classify cases into the best category based on the input predictors. We choose to test an ANN standing alone, without the need of demographical or clinical conditions of subjects used as covariates; so our ANN worked without covariates, and all the inputs were possible predictor factors. Because the computation of speed and step lengths by the IMU software needed the manual input about the definition of the walked distance, these parameters were not taken into account, since we were basing our ANN only on parameters automatically estimated by the wearable device.

### Statistical Analysis

Data were reported in terms of means and standard deviations for the three groups of subjects (healthy subjects, patients who returned, and those who did not return to work). A preliminary analysis of variance was performed, followed by Tukey's *post-hoc* analysis, to highlight gait parameters significantly different among the three groups.

The performances of our feedforward neural network (FFNN) were compared with those of a forward stepwise logistic regression (FSLR), typically used for identifying the prognostic factors of patients with stroke. The normalized importance of input factors evaluated by the FFNN (with 100% as the most possible value) was compared with the *p*-value of those input factors evaluated by logistic regression (with *p* < 0.05 for a statistically significant result: some variables could enter into the model of logistic regression despite a value of *p* > 0.05 because, if removed, the effect was <0.05).

The performances were tested in terms of accuracy (the percentages of correct identifications, given by the sum of true positive and true negative divided by the sample size), sensitivity (the percentages of correct identifications of positive cases: subjects correctly identified as cases on the total number of cases), and specificity (the percentage of correct identification of true negatives: subjects correctly identified as non-case on the total number of non-cases). In the first analysis (identification of patients), we defined the patients as cases and the healthy subjects as non-cases. In the second analysis (identification of not working subjects), all healthy subjects and patients who returned to work were non-cases, whereas patients who did not return to work were cases. Then, in the third analysis (identification of not working patients), only patients' data were analyzed with those who did not return to work classified as cases and those who returned to work as non-cases. The odds ratio and relevant 95% confidence interval (CI_95%_) were computed for the FSLR, whereas the receiver operating characteristic (ROC) curve was computed for the FFNN and the relevant area under the curve was evaluated. For all the statistics, the IBM SPSS Statistic, version 23, was used.

## Results

The gait parameters estimated by the IMU are reported in Table 1. Significant differences were found for cadence and the mean percentage values of gait phases among the three groups of subjects. *Post-hoc* analyses showed that these parameters were significantly different in the group of patients who did not return to work, but not in those who returned to work, with respect to healthy subjects. The trunk obliquity ROM resulted significantly lower in patients who did not return to work and more asymmetric in patients who returned to work.

**Table 1:** Means ± standard deviations of gait parameters estimated by the inertial measurement unit for healthy subjects, patients who returned to work and patients unable to return to work.

**Type of variable**	**Gait parameter**	**Healthy subjects**	**Patients returned to work**	**Patients not returned to work**	***p*-value**
Mean values of gait	Cadence (steps/min)	114 ± 9	109 ± 10	**97** **±** **12**	** <0.001**
parameters	Stance phase (%)	60.7 ± 1.7	60.6 ± 2.5	**64.2** **±** **3.8**	**0.005**
	Swing phase (%)	39.9 ± 1.7	39.4 ± 2.5	**36.8** **±** **2.2**	**0.009**
	1st double support (%)	10.7 ± 1.7	10.6 ± 2.3	**13.1** **±** **2.3**	**0.015**
	2nd double support (%)	10.8 ± 1.7	10.6 ± 2.6	**13.2** **±** **2.2**	**0.013**
	Single support phase (%)	39.2 ± 1.7	39.5 ± 2.6	**37.0** **±** **2.3**	**0.022**
	Tilt ROM (degrees)	6.3 ± 2.1	6.0 ± 1.6	5.6 ± 2.1	0.728
	Obliquity ROM (degrees)	14.5 ± 4.5	9.9 ± 5.4	**7.9** **±** **4.5**	**0.004**
	Rotation ROM (degrees)	18.0 ± 6.3	14.0 ± 5.8	12.5 ± 6.9	0.100
Asymmetry in gait parameters (side-to-side differences)	Stance phase (%)	1.5 ± 2.4	3.0 ± 1.8	5.4 ± 6.3	0.058
	Swing phase (%)	1.5 ± 2.4	3.0 ± 1.8	3.4 ± 3.8	0.205
	1st double support (%)	1.4 ± 1.1	2.0 ± 1.5	1.4 ± 1.5	0.645
	2nd double support (%)	1.5 ± 1.2	2.1 ± 1.3	1.3 ± 1.2	0.453
	Single support phase (%)	1.4 ± 2.3	3.2 ± 1.7	3.3 ± 3.5	0.173
	Tilt ROM (degrees)	0.2 ± 0.3	0.3 ± 0.3	0.3 ± 0.3	0.717
	Obliquity ROM (degrees)	0.2 ± 0.3	**0.6** **±** **0.5**	0.4 ± 0.2	**0.013**
	Rotation ROM (degrees)	0.4 ± 0.3	1.0 ± 0.7	0.8 ± 0.6	0.069

*The last column report the p-values of the analysis of variance performed among the three groups (p-values are reported in bold if statistically significant, whereas data are in bold if post-hoc analysis revealed that they are significantly different from those of healthy subjects)*.

The first analysis tested the capacity of the FFNN to identify the patients with stroke gait with respect to the healthy subjects. The FFNN showed an accuracy of 90.9%, a sensitivity of 93.8%, and a specificity of 88.2% in the identification of patients with stroke. The area under the ROC curve was 0.930. The most important parameters for the FFNN resulted the trunk obliquity ROM (100%) followed by the percentage duration of stance phase (99.6%). When the same investigation was performed using the FSLR, the accuracy in patient identification was 75.8% with a sensitivity of 68.8% and a specificity of 82.4%. The variables entered into the model of the FSLR were the same as those of the FFNN: the trunk obliquity ROM (OR = 0.717, *p* = 0.010, CI_95%_ = 0.56–0.92) and the percentage duration of the stance phase (OR = 1.547, *p* = 0.088, CI_95%_ = 0.94–2.55). The latter one had a not statistically significant effect (*p* = 0.088), but if removed by the model, this effect was significant (*p* = 0.016). These results and those of the further analyses are reported in Table 2.

**Table 2:** Comparisons of the performances (accuracy, sensitivity, and specificity) of feedforward neural network (FFNN) and forward stepwise logistic regression (FSLR) for identification of patients and patients unable to return to work (No-RTW).

**FFNN vs.FSLR**	**Group**	**Healthy subjects and patients**		**Healthy subjects and patients**		**Only patients**	
	**Parameter**	**Patient identification**		**No-RTW identification**		**No-RTW identification**	
	**Model**	**FFNN**	**FSLR**	**FFNN**	**FSLR**	**FFNN**	**FSLR**
Model results	Accuracy	90.9%	75.8%	90.9%	81.8%	93.8%	81.3%
	Sensitivity	93.8%	82.4%	90.0%	87.0%	90.0%	90.0%
	Specificity	88.2%	68.8%	91.3%	70.0%	100.0%	66.7%
Input	Cadence	66.3%	0.768	76.2%	**0.017**	61.0%	0.128
Mean values of Input parameters	Stance phase	**99.6%**	**0.088**	83.0%	0.452	73.1%	0.564
	Swing phase	93.4%	0.877	81.5%	**0.054**	71.1%	**0.026**
	1st double support	84.1%	0.875	**100%**	0.678	65.9%	0.732
	2nd double support	89.7%	0.809	64.8%	0.816	67.1%	0.497
	Single support phase	94.5%	0.789	72.5%	0.902	81.2%	0.497
	Tilt	95.6%	0.312	78.8%	0.223	70.8%	0.985
	Obliquity	**100%**	**0.010**	79.5%	0.561	77.5%	0.454
	Rotation	83.6%	0.892	**88.6%**	0.549	95.0%	0.658
Asymmetry of Input (differences of values)	Stance phase	83.6%	0.757	81.6%	0.166	67.1%	0.280
	Swing phase	90.4%	0.795	80.2%	0.283	62.4%	0.408
	1st double support	91.1%	0.467	84.0%	0.833	**98.8%**	0.805
	2nd double support	91.6%	0.532	76.7%	0.378	**100%**	0.409
	Single support phase	81.2%	0.712	76.0%	0.340	62.9%	0.570
	Tilt	59.7%	0.499	68.4%	0.854	52.8%	0.388
	Obliquity	91.0%	0.083	63.7%	0.649	72.9%	0.354
	Rotation	85.1%	0.080	82.8%	0.711	71.4%	0.894

*Below, the normalized importance of input for FFNN (maximum = 100%, in bold the two highest values) and the p-values of the effect of input for FSLR (in bold if entered into the model because their effect was statistically significant, or if the effect of their removal from the model was significant)*.

The second analysis was focused on the capacity of the FFNN to identify the patients who did not return to work with respect to the entire sample. The accuracy of the FFNN in this identification was 90.9%, resulting from a specificity of 91.3% and a sensitivity of 90%. In fact, the FFNN had only two false positive cases and one false negative case. The area under the ROC curve was 0.978. The variables that mostly contributed to the FFNN were the percentage duration of the first double support phase (100%) and trunk rotation ROM (88.6%). The logistic regression showed an accuracy of 81.8% in this investigation. The variables entered into the model were the cadence (at the first step of regression) and the percentage duration of the swing phase (at the second step). The former entered into the model in the first step and showed a significant effect (*p* = 0.017, OR = 0.87, CI_95%_ = 0.78–0.98), whereas the latter entered into the model in the second step, but with a not significant effect (*p* = 0.054, OR = 0.449, CI_95%_ = 0.20–1.01). It should be specified that despite the effect of percentage duration of the swing phase being not significant, if removed by the model, it had a significant effect (*p* = 0.007).

The third analysis was performed only on the patients. The accuracy of the FFNN was 93.8%, with the same sensitivity as the second analysis (90%) but with a specificity that is even higher (100%). In this analysis, the most important parameter resulted the asymmetry in both the double support phases, followed by the trunk rotation ROM, which was already found as playing a key role for the identification of subjects who did not return to work also in the second analysis. Also, accuracy, sensitivity, and specificity of the FSLR were similar to those found in the second analysis, with the swing phase again found as a variable entered into the model, the only one in this case.

## Discussion

The FFNN showed good performances both in identifying patients with respect to healthy subjects as well as in identifying those patients unable to return to work among all the enrolled subjects. Its performances were higher than those of a classical statistical analysis such as the FSLR. It is noteworthy that both the analyses (FFNN and FSLR) had some analogies in the identification of the parameters that mostly contributed to the outputs (as well as with the results of the preliminary analysis of variance). In both, the identification of patients was based on the percentage duration of the stance phase and on the range of motion of trunk obliquity. The gait phases, and in particular the ratio between stance and swing phases, have been highlighted as fundamental for a harmonic walking because they formed, together with the double support phases, a fractal autosimilar structure of walking (19). The autosimilarity of the ratios between consecutive gait phases is altered in pathological conditions (20, 21), but in physiological conditions, walking allows for an optimization of energy expenditure (21) and an optimal equilibrium between balance and speed (22). In our study, the FSLR showed that a longer stance phase was associated to the identification of patients (OR = 1.547). The trunk kinematics was also reported as fundamental for the upright balance during walking in patients with stroke, being exposed to the risk of fall (23, 24). The inertial measurement of trunk kinematics during walking is probably the most important factor associated to this dynamic balance (11, 12). An excessive obliquity of trunk could be used as a compensation strategy for lower limb deficits in neurological and also neuromuscular diseases (12, 25). It is conceivable that patients who returned to work adopted this strategy to compensate for the affected side, resulting in an asymmetric lateral trunk bending during walking. Conversely, patients unable to return to work showed a lower trunk obliquity range of motion than healthy subjects (OR = 0.717). It was probably due to the reduced walking speed, which reduces the trunk oscillations (10, 11), and to the incapacity to put in action the above-described effective compensation strategy.

The inability to return to work was found associated by the ANN to the first double support phase and to the trunk rotation ROM. In this case, these parameters were different from those identified by analysis of variance as significantly different among groups and by logistic regression, for which cadence and swing phase entered into the model. In this latter case, reduced cadence and reduced limb oscillation phases were found to be associated to patients who did not return to work. This is conceivable with a reduced speed and hence a reduced mobility. For the ANN, the most important parameters associated to not returning to work were those related to the double support phase. This result was in accordance with that of logistic regression: the longer the double support phase, the longer the stance, and the shorter the swing phase (19). But also trunk rotation ROM highly contributed for the ANN, being reduced in patients who are unable to return to work. As well as for trunk obliquity, also rotation could be related to a reduced speed, but its reduction can also be associated with a lack of upper limb oscillations during walking. The contralateral oscillations of the upper limb with respect to the lower limbs are a strategy to stabilize the trunk and the head during walking, counteracting the momentum produced by the lower limbs during their swing phases (12).

The identification of inability to return to work performed only on patients (the third analysis of this study) also confirmed that the FFNN had higher accuracy and specificity than the FSLR, with similar sensitivity. However, caution is needed in the interpretation of these last results given the small sample size of this specific analysis (*n* = 16) with respect to the high number of computed parameters (*n* = 17). In spite of this, this analysis also confirmed a key role of double support phases and trunk kinematics for correct identification performed by the FFNN, whereas the swing phase was confirmed as a variable that should enter into the model of the FSLR.

With respect to logistic regression and general linear models, an artificial neural network has two main advantages. The first one is that the ANN exploits the contribution of each variable that concurs in the identification, whereas in the logistic regression, only those entering into the model do it. On one hand, it could be a complication because the model includes all the inputs, but on the other hand, the accuracy of the output is higher. The second advantage is that each variable can contribute only in a linear manner in the logistic regression and analyses of variance, whereas more complex relationships, also including linear or non-linear interactions, can be taken into account by an ANN. This aspect could be very important: the physiological stance to swing ratio is about 1.618 (19); both a reduction as well as an increment of this ratio could be associated to a pathological walking with a consequent increment of energy expenditure (21). The ANN could intercept this non-linear alteration more than the logistic regression, although the interpretation of the importance of each variable into the ANN is more hidden than as it is in the logistic regression. Other potential disadvantages of the ANN are its sensitivity to the setup of the parameters in its architecture, which may also reduce the repeatability of its optimization process.

This study had some limits; first of all, it should be considered as a pilot study because of the reduced sample size that also led to a reduced number of patients with stroke who returned to work. Another limit is its focus on gait; further analysis may include also upper limb kinematic analysis, cognitive factors, and potential social and environmental barriers into the input of the ANN. At the same time, this study has some strengths such as the innovative approach of combining an IMU and an ANN for determining the possibility to return to work of patients with stroke. The second one is its intrinsic simplicity despite the technologies used. We chose to use only one IMU without any external input (such as spatial distance helpful to compute walking speed or step lengths) and also without any covariate (age or other demographical features, clinical scale scores, and other clinical information were not used). Then, the architecture chosen for the FFNN was relatively simple. The resulting system was standalone.

In conclusion, the wide amount of clinical data today that is easily measurable with wearable technologies needs a powerful computational analysis, such as those provided by artificial neural networks. The hidden layers of artificial intelligences should not scare clinicians, but it is fundamental to provide meaningful information that is helpful for them and especially for patients (26). The integration of machine learning with instrumental movement analysis not only may simplify the assessment of several interdependent parameters (27) but also may provide an evolution of gait analysis allowing for the identification of parameters related to poorly explored fields, such as the return to work and the related quality of life of people affected by long-term disability due to stroke.

## Data Availability Statement

The raw data supporting the conclusions of this article will be made available by the authors, without undue reservation.

## Ethics Statement

The studies involving human participants were reviewed and approved by Independent Ethical Committee of Santa Lucia Foundation. The patients/participants provided their written informed consent to participate in this study.

## Author Contributions

MI and MP: conceptualization. EC, SPe, and MP: data collection. MI: data analysis and original draft preparation. GM, GA, and SPa: review and editing of the draft. MI, MP, SPa, and GA: supervision. MP, MI, and BP: project administration and funding acquisition. All authors have read and agreed to the published version of the manuscript.

## Conflict of Interest

The authors declare that the research was conducted in the absence of any commercial or financial relationships that could be construed as a potential conflict of interest.
